# Neuropsychiatric symptoms and neuroimaging‐based brain age in mild cognitive impairment and early dementia: A multicenter study

**DOI:** 10.1111/pcn.13777

**Published:** 2025-01-17

**Authors:** Daichi Sone, Iman Beheshti, Kenji Tagai, Hiroshi Kameyama, Emi Takasaki, Tetsuo Kashibayashi, Ryuichi Takahashi, Kazunari Ishii, Hideki Kanemoto, Manabu Ikeda, Masahiro Shigeta, Shunichiro Shinagawa, Hiroaki Kazui

**Affiliations:** ^1^ Department of Psychiatry Jikei University School of Medicine Tokyo Japan; ^2^ Department of Human Anatomy and Cell Science, Rady Faculty of Health Sciences, Max Rady College of Medicine University of Manitoba Winnipeg Manitoba Canada; ^3^ Nishi‐Harima Dementia‐Related Disease Medical Center Hyogo Prefectural Rehabilitation Hospital at Nishi‐Harima Tatsuno Japan; ^4^ Department of Radiology Kindai University Faculty of Medicine Osaka Japan; ^5^ Department of Psychiatry Osaka University Graduate School of Medicine Osaka Japan; ^6^ Department of Neuropsychiatry, Kochi Medical School Kochi University Kochi Japan

**Keywords:** dementia, machine learning, magnetic resonance imaging, mild cognitive impairment, neuropsychiatric symptoms

## Abstract

**Aim:**

Despite the clinical importance and significant social burden of neuropsychiatric symptoms (NPS) in dementia, the underlying neurobiological mechanism remains poorly understood. Recently, neuroimaging‐derived brain‐age estimation by machine‐learning analysis has shown promise as an individual‐level biomarker. We investigated the relationship between NPS and brain‐age in amnestic mild cognitive impairment (MCI) and early dementia.

**Methods:**

In this cross‐sectional study, clinical data, including neuropsychiatric inventory (NPI), and structural brain MRI of 499 individuals with clinical diagnoses of amnestic MCI (*n =* 185), early Alzheimer's disease (AD) (*n =* 258) or dementia with Lewy bodies (DLB) (*n =* 56) were analyzed. We established a brain‐age prediction model using 694 healthy brain MRIs and a support vector regression model and applied it to the participants' data. Finally, the brain‐predicted age difference (brain‐PAD: predicted age minus chronological age) was calculated.

**Results:**

All groups showed significantly increased brain‐PAD, and the median (IQR) brain‐PAD was 4.3 (5.4) years in MCI, 6.3 (6.2) years in AD, and 5.0 (6.5) years in DLB. The NPI scores were subdivided into the following four categories: (i) Agitation and Irritability, (ii) Depression and Apathy, (iii) Delusions and Hallucinations, and (iv) Euphoria and Disinhibition. We found a significantly positive correlation between brain‐PAD and the depression/apathy factor (Spearman's rs = 0.156, FDR‐corrected *P* = 0.002), whereas no significance was shown for the other NPS factors.

**Conclusion:**

Higher brain‐age may be associated with depression and apathy symptoms presented in MCI to early dementia stages, and brain‐age analysis may be useful as a novel biomarker for the assessment or monitoring of NPS.

Neuropsychiatric symptoms (NPS) in dementia – also known as behavioral and psychological symptoms of dementia (BPSD) – including hallucinations, delusions, depression, and apathy are clinically significant symptoms that reduce an individual's quality of life and increase the burden on caregivers.^1^, ^2^ NPS can emerge from the mild cognitive impairment (MCI) stage and follow various trajectories.^3^ There is still much debate about the optimal pharmacological and non‐pharmacological interventions for NPS,^4^ and many clinical issues regarding NPS thus remain to be resolved.

The neural mechanisms underlying NPS in dementia are also insufficiently understood.^5^ Although neuroimaging plays a significant role in diagnosing Alzheimer's disease (AD) as an established modality,^6^ the neuroimaging correlates of NPS are quite diverse. According to a systematic review,^7^ although delusions, apathy, and depression were the symptoms most likely to correlate with brain imaging abnormalities (particularly in the frontal or temporal lobes), there was huge diversity in the findings, including correlations regarding other symptoms or brain regions, e.g. subcortical areas. It is also suggested that NPS is not always associated with atrophy.^8^ More recently, a single‐center study using *in vivo* tau imaging reported NPS domain‐specific regions with tau deposition,^9^ but a neuropathological investigation failed to find any specific associations with NPS domains, despite a general relationship between Braak stages and NPS.^10^

Given the heterogeneity of the regional brain changes associated with NPS, there may be value in seeking biomarkers from other perspectives beyond local brain alterations. In other words, it may be difficult to simply attribute NPS in dementia to specific brain regional abnormalities. Abnormal aging processes have received attention in the past decade, particularly in the field of age‐related diseases, including dementia.^11^ Advances in machine learning have made it possible to construct systems that can be used to estimate the age of an individual's brain based on neuroimaging features, and neuroimaging‐based brain‐age estimation is highly promising as a personalized biomarker in neurological and psychiatric disorders.^12^ Increased brain‐age in AD has been consistently reported, and it has also been suggested that brain‐age could be used to predict the conversion from MCI to AD^13^ or identify cognitively unimpaired amyloid‐positive individuals.^14^ However, the relationship between brain‐age and NPS has not been well studied.

We hypothesized that brain‐age in the MCI and AD diagnostic groups may be associated with NPS; in particular, brain‐age may influence an individual's risks of delusions, apathy, and/or depression because these neuropsychiatric symptoms seem to be more closely associated with brain morphological abnormalities.^7^ If a relationship between brain‐age and NPS is established, brain‐age could be clinically useful as a surrogate marker for monitoring and risk prediction. Such a relationship may also suggest that some protective factors or interventions reported to reduce brain‐age values might be effective in treating NPS. In addition, when considering possible prevention or intervention measures, it would be better to focus on the early stages rather than the progressed stages. We thus investigated the relationship between brain‐age and NPS in patients with amnestic MCI and early dementia in a cross‐sectional study.

## Methods

### Patients

This study was a part of a multicenter project in Japan, ‘The Japan multicenter study: Behavioral and psychological symptoms Integrated Research in Dementia (J‐BIRD)’.^15^ The structural neuroimaging and clinical data were collected during the period April 2015 through December 2021 from three specialized centers for psychiatry and neurology: Osaka University, Kochi University, and Jikei University. The patients were suspected of having cognitive dysfunctions and were subsequently clinically diagnosed with amnestic MCI, AD, or dementia with Lewy bodies (DLB) with a Clinical Dementia Rating (CDR) of 0.5–1.0.

All patients underwent a careful clinical interview, standard neurocognitive tests including the Mini‐Mental State Examination (MMSE) and CDR, an evaluation of NPS by the Neuropsychiatric Inventory (NPI) (12‐item version),^16^ and high‐resolution brain structural MRI. When needed, brain perfusion imaging using 123I‐N‐isopropyl‐p‐iodoamphetamine (IMP) single‐photon emission computed tomography (SPECT) was also performed. All of the clinical evaluations were conducted by board‐certified dementia specialists at each facility. The clinical diagnoses were based on the clinical criteria for probable AD,^17^ the Petersen's criteria for amnestic MCI,^18^ and the consensus criteria for probable DLB.^19^

Since brain‐age is usually correlated with cognitive dysfunction itself,^12^ our present analyses included only patients with MCI or mild dementia with relatively similar cognitive function with CDR values ranging from 0.5 to 1, in order to expose the association between NPS and individual brain‐age. The exclusion criteria were as follows: (*i*) a present or past diagnosis of cerebrovascular disease (CVD) confirmed by MRI, (*ii*) a history of a neurological, psychiatric, or physical disorder that may influence cognitive decline other than dementia, and (*iii*) any abnormal findings other than brain atrophy on MRI.

Although the clinical diagnosis of MCI, AD, and DLB was carefully performed by experts, this study was conducted in a clinical setting and did not involve more confirmatory testing for neuropathology, e.g. amyloid PET. In addition, it is well known that individuals with DLB frequently present AD‐related changes in autopsy neuropathological examinations.^20^ The present MCI group was also expected to include a mix of patients with various neuropathological changes. We thus decided to analyze all categories of AD, DLB, and amnestic MCI together, as doing so may lead to the identification of common associations between NPS and brain aging among these categories.

This study was conducted at Tokyo Jikei University Hospital, Kochi Medical School Hospital, and Osaka University Hospital. The ethics committees at each facility, namely Ethical Review Board of Kochi Medical School (the main institute, approval no. 2021–92), Jikei University Ethics Committee, and Osaka University Research Ethics Committee, approved the investigation based on the ethical standards outlined in the Helsinki Declaration, and the need for patient informed consent was waived due to the use of anonymized data.

### Assessment of NPS levels

Each patient's NPS was evaluated using the Japanese version of NPI,^16^ which includes the original 10 items, i.e. delusions, hallucinations, agitation, depression (dysphoria), anxiety, euphoria, apathy, disinhibition, irritability, and aberrant motor behavior as well as two additional items, i.e. night‐time behavior disturbances and appetite/eating abnormalities. The degree of each symptom of NPS was assessed by the composite score obtained by multiplying the frequency and severity of the NPI subscales.^15^ Thereafter, to extract the principal factors of the NPS subdomains, we performed a principal component analysis for the 12 NPI composite scores, and the factor scores were calculated. To simplify the factor loadings and enhance interpretability, we used a varimax rotation after confirming no strong correlations between component factors. We extracted the components with the initial eigenvalues >1, because these components have at least one variable's worth of information.

### MRI acquisition and processing

All participants underwent 3D T1‐weighted brain MRI scanning with one of the following protocols: (i) Jikei (Siemens 1.5T, Magnetom Avanto): repetition time (TR)/echo time (TE), 1300 ms/ 3 ms; TI, 800 ms; flip angle, 15°; voxel size, 0.9 × 0.9 × 0.9 mm, (ii) Kochi (Philips 3T, Ingenia): TR/TE, 7.1 ms/3.3 ms; TI, 824 ms; flip angle, 9°; voxel size, 1.14 × 1.14 × 1.2 mm, (iii) Osaka‐1 (GE 1.5T, Signa Excite HD): TR/TE, 12.6 ms/4.2 ms; inversion time (TI), 400 ms; flip angle, 15°; voxel size, 0.9 × 0.9 × 1.4 mm, (iv) Osaka‐2 (GE 1.5T, Signa Explorer): TR/TE, 11 ms/4.2 ms; TI, 400 ms; flip angle, 15°; voxel size, 0.9 × 0.9 × 1.4 mm.

The pre‐processing of MRI data was performed using the voxel‐based morphometry (VBM) technique, which was implemented in the CAT12 Toolbox (http://www.neuro.uni-jena.de/cat/) with a default set of parameters in the Statistical Parametric Mapping software program (SPM12: http://www.fil.ion.ucl.ac.uk/spm/software/spm12/) running on Matlab2021a (MathWorks, Natick, MA, USA). Using the VBM method, we constructed density images of gray matter (GM), white matter, and cerebrospinal fluid from each T1‐weighted MRI scan. Considering the presence of white‐matter hyperintensity loads in some of the patients, we used only GM images (i.e. cortical and subcortical GM features). The quality of the MRI processing and segmentation was visually confirmed.

### Brain‐age estimation model

The flowchart is shown in the Suppl. File S1. We used 3DT1‐weighted brain MRI scans from 694 healthy controls (HCs) (age range 40–94 years, mean age 49.75 ± 18.96 years, 56% males) to train and validate our brain‐age prediction model. To build an accurate brain‐age prediction model, it was necessary to cover a wider range of brain ages that our patient cohort might exhibit, as the brain age estimates for the patient group should fall within the age range of the HCs group. We thus incorporated healthy subjects >40 years of age, and the mean age of the HCs was lower than that of the patient group. The age distribution of our datasets across the HCs and patients is displayed in the Suppl. File S2.

These scans of HCs were obtained from the Open Access Series of Imaging Studies (OASIS: https://www.oasis-brains.org/), the IXI Dataset (https://brain-development.org/ixi-dataset/), and the Parkinson's Progression Markers Initiative (PPMI) database (https://www.ppmi-info.org/). The demographic data of each HC database are shown in the Suppl. File S2. All of the HCs in these databases had no cognitive impairment or neurological diseases. We randomly selected 90% of the HCs to build our brain‐age prediction model, and the remaining 10% were used as a sample for validation.

The spatial normalization process of the brain MRI scans was the same as that used for the patient group. The preprocessed GM images underwent smoothing with a 4‐mm Gaussian kernel before being re‐sampled to an 8‐mm isotropic spatial resolution, yielding a volume of 3747 voxels. We used a linear kernel support vector regression (SVR) algorithm to estimate the brain‐ages. This specific algorithm was chosen because of its proven effectiveness and frequent use in previous brain‐age studies.^12^, ^21^ Our prediction model incorporated voxel intensities of GM along with sex, scanner vendor, field strength, and total intracranial volume (TIV) calculated by CAT12 as independent variables; the actual patient age was the dependent variable. Scanner‐related information was incorporated into the model to minimize potential discrepancies between the different scanners. To ensure unbiased brain‐age values, we used a validated bias adjustment scheme.^22^ Ten‐fold cross‐validation was applied to assess the prediction performance in the training set. The accuracy of the prediction was evaluated in the training and validation sets using the mean absolute error (MAE) and coefficient of determination (*R* ^2^) between the chronological and predicted brain‐ages of the HCs.

The established brain‐age prediction model was applied to the processed GM images of the patients, and finally the brain‐predicted age difference (brain‐PAD: predicted age minus chronological age) was calculated for each patient.

### Statistical analyses

The statistical analyses were performed using SPSS software ver. 25.0. Categorical variables were compared using χ^2^‐tests. The distributions of continuous variables were tested by the Shapiro–Wilk test, and we observed non‐parametric distributions of almost all of the parameters. To compare the brain‐PAD values between the patient categories and HCs, we thus used the Kruskal–Wallis test followed by the post‐hoc Dunn test. To assess the relationships between NPS and brain‐age, Spearman's rank correlation test was used to analyze correlations of the patients' brain‐PAD with each factor score of NPI. A *P*‐value <0.05 with false‐discovery rate (FDR) correction for the number of correlation analyses, i.e. the number of subdomains, was deemed significant to correct for the multiple analyses.

## Results

### Demographics

A total of 499 patients with MCI or mild dementia (MCI, *n* = 185; AD, *n* = 258, DLB, *n* = 56) were enrolled. As shown in Table 1, there was no significant difference in age or sex among the categories, although the AD group tended to have a higher proportion of females. Regarding the total NPI scores, the DLB group showed significantly higher values compared to the other two groups (both *P* < 0.001), and the corresponding difference between the AD and MCI groups was also significant (*P* = 0.003).

**Table 1:** Demographics of the participants in each clinical diagnostic category in this study

	MCI	AD	DLB	*P*‐value
*n*	185	258	56	
Males:females	79:106	88:170	27:29	0.059
Median age, years. (IQR)	78 (10)	79 (10)	79 (8)	0.100
Median MMSE, points (IQR)	26 (3)	21 (4)	22.5 (5)	<0.001
CDR, *n*	0.5, 180; 1.0, 5	0.5, 132; 1.0, 126	0.5, 31; 1.0, 25	<0.001
Median total NPI score (IQR)	4 (11)	7 (13)	20 (19)	<0.001
Median brain‐PAD (IQR)	4.3 (5.4)	6.3 (6.2)	5.0 (6.5)	<0.001

AD, Alzheimer's disease; brain‐PAD, brain predicted age difference; DLB, dementia with Lewy bodies; MCI, mild cognitive impairment; MMSE, Mini‐Mental State Examination; NPI, Neuropsychiatric Inventory.

### NPS domains

The results of the principal component analysis for the NPI subdomains are presented in Table 2. We observed four principal factors: Agitation and Irritability (Factor 1), Depression and Apathy (Factor 2), Delusions and Hallucinations (Factor 3), and Euphoria and Disinhibition (Factor 4).

**Table 2:** Results of the principal component analysis, the NPI subscales, and the correlation with brain‐PAD

	Factor 1: Agitation & Irritability	Factor 2: Depression & Apathy	Factor 3: Delusions & Hallucinations	Factor 4: Euphoria & Disinhibition
Eigenvalue	1.896	1.765	1.748	1.451
Variance explained, %	15.8	14.7	14.6	12.1
Subscales				
Delusions	0.082	−0.129	0.507	−0.109
Hallucinations	−0.149	−0.111	0.583	0.016
Agitation	0.491	−0.137	0.022	−0.093
Depression	0.240	0.301	−0.161	−0.163
Anxiety	−0.018	0.197	0.080	0.155
Euphoria	−0.083	−0.098	−0.065	0.642
Apathy	0.065	0.441	−0.040	−0.297
Disinhibition	0.276	−0.137	−0.084	0.297
Irritability	0.412	−0.029	−0.061	−0.102
Aberrant motor behavior	0.065	0.049	0.086	0.137
Night‐time behavior disturbances	−0.206	0.197	0.116	0.328
Appetite and eating abnormalities	−0.204	0.527	−0.140	0.101
Correlation with brain‐PAD				
Spearman's ρ	0.077	**0.156**	−0.058	−0.026
*P*‐value, FDR‐corrected	0.170	**0.002**	0.257	0.568
*P*‐value, uncorrected	0.085	**0.00047**	0.193	0.568

*Note*: Bold font: significant results.

brain‐PAD, brain predicted age difference; FDR, false discovery rate; NPI, Neuropsychiatric Inventory.

### Brain‐age prediction model

**Fig. 1:**
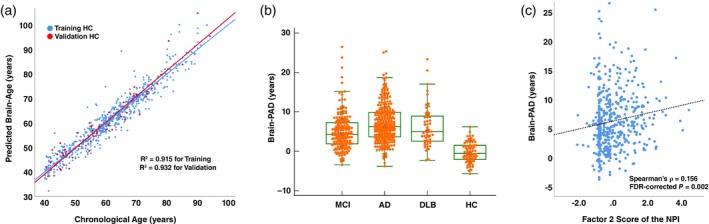
(a) The relationship between predicted brain‐age by neuroimaging and chronological age in the healthy controls (HCs). (b) The brain predicted age difference (brain‐PAD) in the HCs and the patients in each diagnostic category. (c) The correlation between brain‐PAD and the depression and apathy score (factor 2) of the Neuropsychiatric Inventory (NPI) in the patients.

As depicted in Figure 1a, our brain‐age prediction model provided the following values: MAE = 2.55 years, R^2^ = 0.915 in the training set, and MAE = 2.14 years, R^2^ = 0.932 in the validation set. These metrics are comparable with those of similar studies^12^, ^21^ and indicate the model's high accuracy in estimating brain age. No significant age dependency was observed in the training set or the validation set. The mean Brain‐PAD scores in the training and validation sets were 0 years and −0.26 years, respectively.

### The Brain‐PAD in each clinical diagnostic category

Although this study's main purpose was not to detect group‐level alterations of brain‐PAD values in each category, we identified significantly increased brain‐PAD values in all diagnostic categories compared to the HC group (all *P* < 0.001, Table 1, Fig. 1b). The AD group showed significantly higher brain‐PAD values compared to the MCI group (*P* < 0.001). The results of the other comparisons between patient groups were not significant. The brain‐PAD did not correlate with chronological age of patients (Spearman's ρ = 0.056, *P* = 0.210) nor differ between males and females (*P* = 0.167, Mann–Whitney U test). As expected, MMSE was correlated with brain‐PAD (Spearman's ρ = −0.271, *P* < 0.001).

Between the four MRI scanners, there was no significant difference in brain‐PAD results, and the severity of cognitive impairment was also not significantly different in terms of the MMSE or CDR (Suppl. File S2).

### The relationships between NPS and brain‐PAD

As shown in the Table 2 and Figure 1c, our analyses revealed a significant positive correlation between the brain‐PAD and Factor 2 of the NPI score, i.e. Depression and Apathy (Spearman's ρ = 0.156, FDR‐corrected *P* = 0.002). The other factors were not significantly correlated with the brain‐PAD scores. We also performed an additional analysis to investigate correlations of the brain‐PAD with each raw score of NPI subscales, in which apathy was most strongly associated with higher brain‐age (Suppl. File S3).

## Discussion

To the best of our knowledge, this is the first study to examine the relationships between neuroimaging‐derived brain‐age values and NPS in individuals with MCI or early dementia. NPS has been subclassified into four domains: agitation and irritability, depression and apathy, delusions and hallucinations, and euphoria and disinhibition, and of these, abnormal brain aging was associated with the depression and apathy factor, while no significant relationships were observed for the other three domains. Our results suggest that those presenting depression or apathy symptoms tend to have older brain, which may provide the potential utility of a brain‐age evaluation as a novel individual‐level biomarker for the risk assessment and/or monitoring of NPS, particularly depressive and apathy symptoms, in elderly patients with cognitive impairment.

Applications of artificial intelligence have enabled remarkable progress in brain research, and many studies have investigated the relationships between brain aging and AD and cognitive dysfunction.^12^ It is known that the brain‐age increases by 5–9 years in AD,^23^, ^24^ which is consistent with our current results (Fig. 1b). An increase in brain‐age is also known to predict conversion to AD in individuals with MCI^13^ and to discriminate the presence/absence of amyloid deposition in cognitively unimpaired elderly subjects.^14^ Although brain‐age alterations in DLB have not been well investigated, it was reported that Parkinson's disease with dementia showed an increase in brain‐age by approximately 3.5 years.^25^ Thus, because of the strong associations between dementia and aging, the brain‐age prediction system by artificial intelligence has been widely studied and is a highly promising individual‐level biomarker in the field of dementia.

Compared to cognitive dysfunction, the neurobiological bases of NPS (or BPSD) are poorly understood,^26^ despite its significant burden on patients and caregivers.^1^ As noted, neuroimaging studies have obtained a remarkable heterogeneity of results, particularly in terms of brain regions,^7^ and we thus focused on an aging biomarker beyond local changes. Apart from neuroimaging findings, genetic studies have suggested the involvement of serotonin transporters in the NPS of individuals with neurodegenerative diseases.^27^ A neuropathological study described serotonergic and noradrenergic abnormalities in Lewy body disease with delusion or depression.^28^ Neurotransmitters may therefore play an important role in NPS. However, another recent study with a larger community‐based autopsy cohort failed to identify any specific pathological alterations in NPS.^10^ Further efforts are thus necessary to address the heterogeneity among study findings and tackle this challenging topic.

In the present cohort, the brain‐age to be higher in the patients with greater depression and apathy. It has been reported that depression can precede and possibly accelerate cognitive decline in AD.^29^, ^30^ Apathy is also an important symptom and is the most prevalent symptom in AD^31^; apathy also worsens functional disability and the caregiver burden.^32^, ^33^ Compared with other neuropsychiatric symptoms, these symptoms have been relatively consistently reported with neuroimaging alterations in which the frontal regions, particularly the anterior cingulate gyrus and orbitofrontal cortex, were mainly involved.^7^ Our findings on brain‐age may reflect these cerebral alterations. In recent years, intrinsic‐connectivity network abnormalities including the anterior cingulate cortex (ACC) and orbitofrontal cortex (OFC) have been suggested as a potential mechanism for apathy,^5^ which may have affected the abnormal brain‐aging process in our cohort. Further studies of the relationship between neural networks and brain‐age are warranted.

In addition, while our principal component analysis categorized depression and apathy into the same parameter, there has been a discussion about the similarity and difference between these two symptoms. Typically, apathy mainly affects volition, and depression is characterized by mood problems, although they share several symptoms.^34^ Our additional analysis identified the strongest correlation of brain‐PAD with apathy and no significance with depression (Suppl. File S3), which would suggest the importance of apathy. In fact, this discordance is consistent with previous findings. Apathy is reported to predict onset of dementia in people with MCI^35^ and to be associated with beta‐amyloid deposition.^36^ Moreover, according to a recent longitudinal study, apathy worsens with the clinical course in dementia and relates with worse clinical consequences independent of depression.^37^ Thus, our results may also accord with the recent focus on apathy as the treatment target in dementia.^34^

It is also known that brain‐age can be affected by various lifestyle‐related factors in the elderly.^12^, ^38^, ^39^ Several adverse factors, e.g. diabetes and alcohol use, are consistently reported to have negative effects on brain‐age, but several protective factors have also been proposed, including music composition,^40^ long‐term meditation,^41^ and life satisfaction.^38^ Whether brain‐age improvement based on these findings (i.e. by promoting protective factors and preventing adverse factors) is effective in improving apathy or depression as neuropsychiatric symptoms in dementia needs to be further investigated. However, as an initial step, our findings may provide some insight into the exploration of novel approaches to NPS in dementia beyond just assessment findings and biomarker monitoring.

Our categorization of NPI subdomains based on the principal component analysis (Table 2) seems consistent with past literature.^42^, ^43^ In fact, according to a comprehensive review,^42^ NPS was generally categorized into affective symptoms, psychosis, hyperactivity, and euphoria. On the other hand, however, it is actually difficult to identify reliable and discrete factors of NPS in dementia. The factor structure of NPS can vary over time,^44^ and a relatively low concordance in the composition of NPI clusters was also suggested.^45^ Thus, the challenging nature of measuring distinct factors of the NPS should be considered in interpreting the results of our study. We did not find any associations of brain‐age with the NPS domains other than Depression and Apathy, i.e. Agitation and Irritability, Delusions and Hallucinations, and Euphoria and Disinhibition (Table 2). These NPS symptoms were generally less associated with brain morphological alterations, except for delusion,^7^ and we thus may need more advanced or other methodologies than the current structural neuroimaging to elucidate the mechanisms underpinning these NPS symptoms.

The strengths of the current study may include the medium size of the samples, the detailed clinical evaluations of NPS by experts, the advanced neuroimaging analysis by machine‐learning, and the special focus on differences in brain‐age. However, several limitations should be noted. First, our cross‐sectional design cannot answer questions about causality. Second, we included brain MRI data from different scanners in the patient cohort and HCs, which might have affected the brain‐age parameters as a confounder. To reduce such effects, we applied several statistical corrections and consequently observed similar brain‐PAD values across all of the scanners (Suppl. File S4). In fact, the CAT 12 processing has been suggested to mitigate certain site‐specific effects by employing a standardized pre‐processing pipeline across all datasets, particularly through the application of the DARTEL registration strategy.^46^ In addition, the T1‐weighted MRI‐based brain age estimation model has been cross‐validated with reference samples to ensure its accuracy and stability across various MRI scanners. This model has demonstrated significant reliability and generalizability, even when applied to data collected from different scanners and field strengths.^11^

Third, the brain‐age is known to be affected by cognitive dysfunction itself, which might have affected the results beyond the effect of NPS. Because of the non‐parametric distributions, we decided to analyze the cognitively homogeneous group with CDR = 0.5–1, rather than statistical correction by covariate analysis, even though this approach may not fully address the potential confounding problems. It is also important to note that the current study only focused on early cases and the results may not be generalized to progressed dementia. The relationship between NPS and severity of cognitive dysfunction is complex; past studies reported heterogeneous results, such as significant correlations in frequency,^47^ lack of association,^48^ or predictability.^49^ To overcome the limitations of symptom‐based clinical evaluations and reveal the complexity of NPS and cognitive dysfunction, neuroimaging studies may be expected to provide objective biomarkers and elucidate neural biomechanisms. Further research would be warranted to include more comprehensive and longitudinal data of patients with various levels of cognitive dysfunction.

Fourth, we analyzed all of the patients, i.e. amnestic MCI, AD, and DLB together, which may have failed to detect pathology‐specific findings. However, as noted, this study aimed at clinical settings and lacked confirmatory examinations for pathology, and mixed pathologies in each clinical diagnostic category would be expected. With the exclusion of patients with DLB, the correlation between brain‐PAD and Factor 2 was also significant (Spearman's ρ = 0.147, *P* = 0.002). In fact, since amnestic symptoms are also prominent in prodromal DLB, clinical misdiagnoses between AD and DLB are common, particularly for late‐onset DLB.^50^, ^51^ Thus, we decided to analyze all patients to identify common mechanisms of NPS in aging.

Moreover, despite the statistical significance (FDR *P* = 0.002), the correlation coefficient between the brain‐PAD and NPS was relatively weak (ρ = 0.156). The biological process of aging is complex and may vary in each subject; thus, at present, it is difficult to use brain‐age as a single biomarker for NPS, and the use of brain‐age would need to be combined with various other clinical indicators. Finally, the possible effects of unevaluated or unknown confounders, e.g. medications and physical diseases, should be incorporated into a careful interpretation of our present findings. Further comprehensive and longitudinal studies may elucidate the complex effects of brain aging on NPS, plus causal relationships.

## Conclusion

The results of our analyses demonstrated a relationship between abnormal brain‐age values and depression/apathy symptoms as a part of NPS in patients with MCI and early dementia. Neuroimaging‐derived brain‐age may be useful for better pathophysiological understanding and clinical assessments of NPS in persons with cognitive impairment.

## Disclosure statement

Manabu Ikeda is an Editorial Board member of Psychiatry and Clinical Neurosciences and a co‐author of this article. To minimize bias, they were excluded from all editorial decision‐making related to the acceptance of this article for publication.

## Author contributions

Conception and design of the study: SS, HKazui, Acquisition and analysis of data: DS, IB, KT, HKameyama, ET, TK, RY, KI, HKanemoto, MI, MS, SS, HKazui, Drafting the manuscript or figures: DS, IB.

## Supporting information


**File S1.** The establishment and applications of the brain‐age estimation model.
**File S2.** The age distribution of our datasets across the HCs and patients and the demographic data of each HC database.
**File S3.** Binary correlations of brain‐PAD with each raw score of NPI subscales.
**File S4.** Scanner differences in terms of demographics and brain‐PAD.

## Data Availability

Data not included in the article will be made available from the corresponding author to qualified researchers on reasonable request subject to ethics approval.
