# Supplementary material for: Neuropsychiatric symptoms and neuroimaging‐based brain age in mild cognitive impairment and early dementia: A multicenter study
Source: Psychiatry Clin Neurosci. 2025 Jan 17;79(4):158–64. doi: 10.1111/pcn.13777 (PMC11962355; doi:10.1111/pcn.13777)

Supplementary file S1. The establishment and applications of the brain-age estimation model

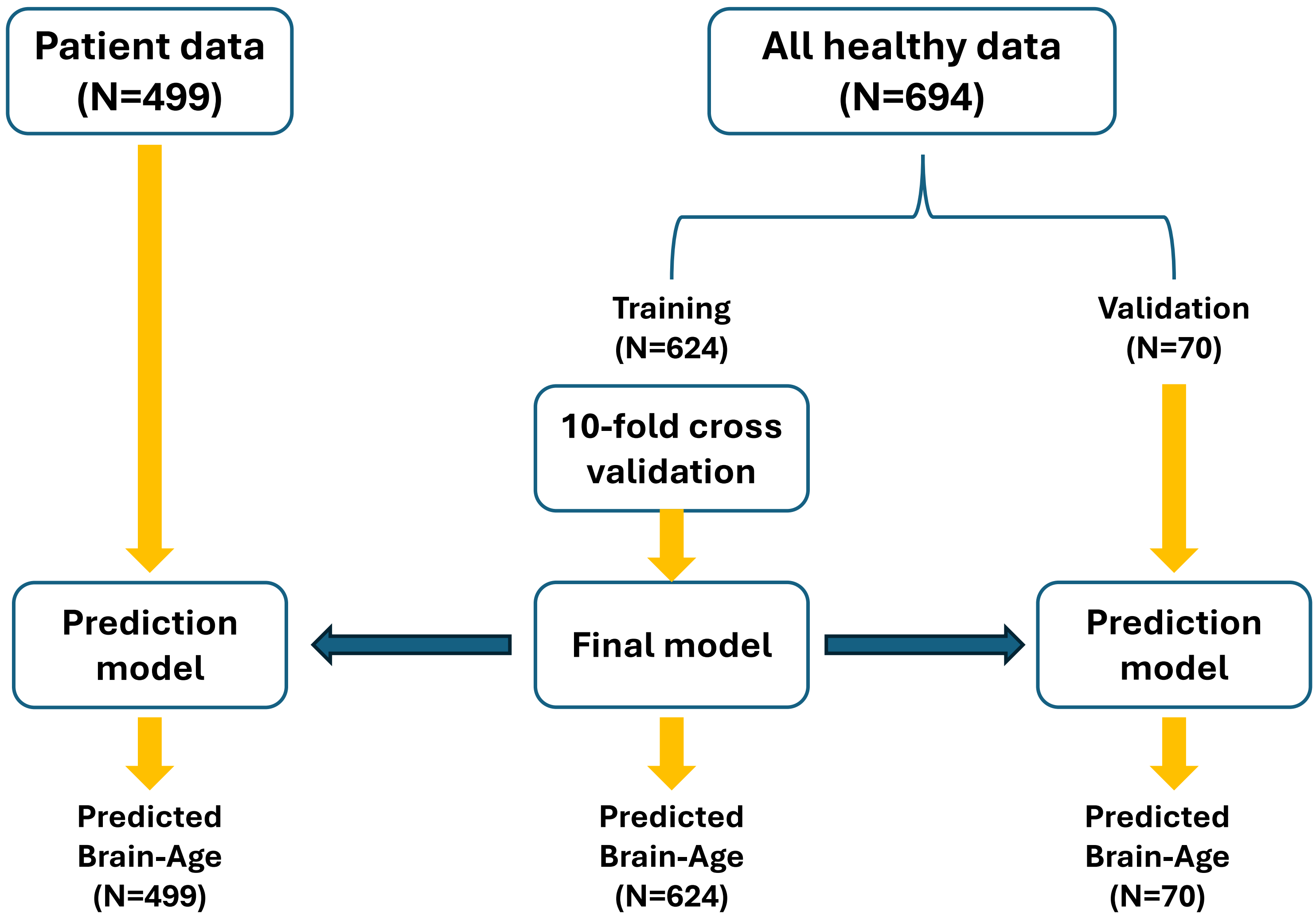

Supplementary file S2. The age distribution of our datasets across the HCs and patients and the demographic data of each HC database.

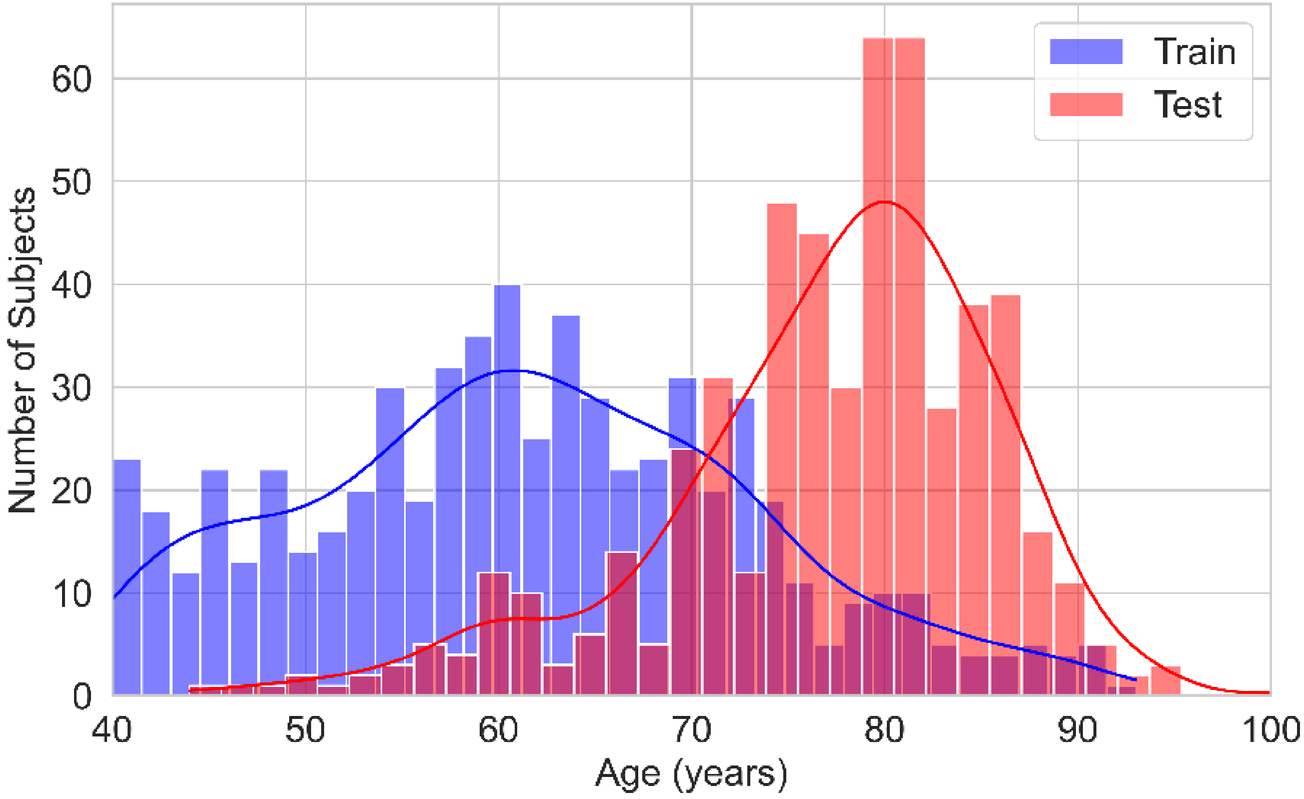

Dataset	N	Mean age	min	max	std	Female	1.5T scanner
IXI	363	58.9	40.0	86.3	10.6	40%	64%
OASIS	162	65.7	40	94	14.8	29%	100%
PPMI	169	61.7	40.2	82.7	9.6	64%	34%

Supplementary file S3. Binary correlations of brain-PAD with each raw score of NPI subscales.

Subscales	Spearman's ρ	p-val. (uncorrected)	Subscales	Spearman's ρ	p-val. (uncorrected)
Delusions	0.004	0.927	**Apathy**	**0.126**	**0.005**
Hallucinations	0.010	0.827	Disinhibition	0.050	0.270
Agitation	0.057	0.203	Irritability	0.074	0.101
Depression	0.051	0.254	Aberrant motor behavior	0.053	0.233
**Anxiety**	**0.101**	**0.025**	Night-time behavior disturbances	0.025	0.571
Euphoria	0.033	0.464	**Appetite and eating abnormalities**	**0.101**	**0.024**

* Bold font denotes uncorrected $p<0.05$

Supplementary file S4. Scanner differences in terms of demographics and brain-PAD.

	Scanner 1 (Jikei)	Scanner 2 (Kochi)	Scanner 3 (Osaka-1)	Scanner 4 (Osaka-2)	p-val.
N	117	111	222	49	
M:F	59:58	34:77	79:143	22:27	0.009
median age (IQR)	81 (9)	76 (11)	78 (9)	78 (12)	0.001
median MMSE (IQR)	24 (7)	23 (6)	23 (5)	23 (6)	0.241
CDR (N)	0.5 = 82, 1.0 = 35	0.5 = 68, 1.0 = 43	0.5 = 163, 1.0 = 59	0.5 = 30, 1.0 = 19	0.088
median total NPI score	4 (10)	7 (15)	8 (14)	8 (21)	0.006
median brain-PAD	6.0 (6.9)	4.8 (4.8)	5.7 (6.7)	5.5 (5.8)	0.069
Diagnosis	54MCI, 59AD, 4DLB	19MCI, 79AD, 13DLB	91MCI, 99AD, 32DLB	21MCI, 21AD, 7DLB	<0.001

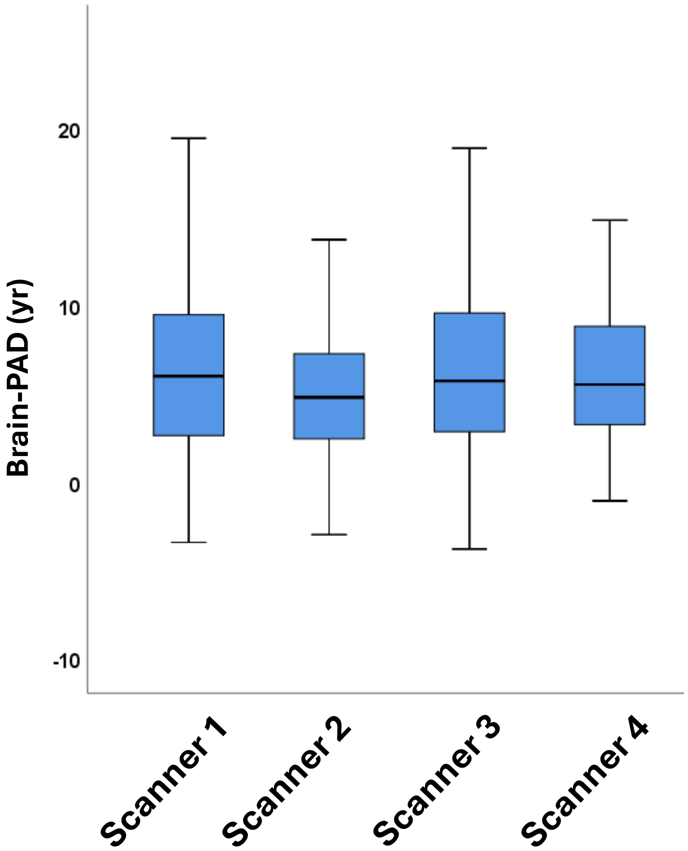

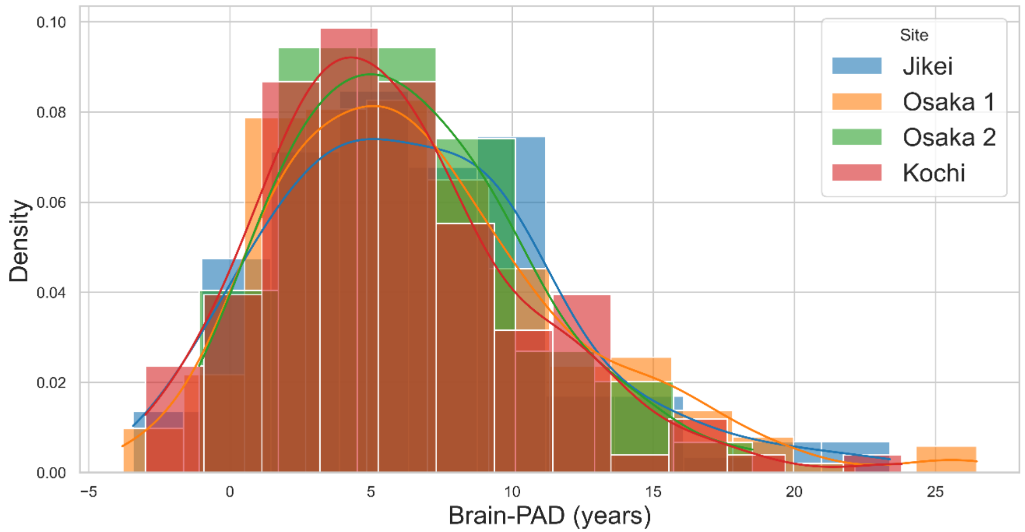

Supplement: Supplementary file 1 — File S1. The establishment and applications of the brain‐age estimation model. File S2. The age distribution of our datasets across the HCs and patients and the demographic data of each HC database. File S3. Binary correlations of brain‐PAD with each raw score of NPI subscales. File S4. Scanner differences in terms of demographics and brain‐PAD. [file PCN-79-158-s001.pdf]
